# Chronic Myeloid Leukemia Presenting as Non-cirrhotic Portal Hypertension

**DOI:** 10.7759/cureus.40692

**Published:** 2023-06-20

**Authors:** Rinkle R Gemnani, Avinash Parepalli, Sunil Kumar, Sourya Acharya, Samarth Shukla

**Affiliations:** 1 Department of Medicine, Jawaharlal Nehru Medical College, Wardha, IND; 2 Department of Pathology, Jawaharlal Nehru Medical College, Wardha, IND

**Keywords:** chronic myeloid leukemia (cml), myeloproliferative neoplasm, jak-2, portal hypertension, cirrhosis

## Abstract

Non-cirrhotic portal hypertension (NCPH) is a poorly understood condition attributed to various causes in the absence of liver cirrhosis. One of the important and rare conditions leading to NCPH is myeloproliferative neoplasms and blood coagulation abnormalities, which infiltrate the liver cells leading to stasis and raised sinusoidal pressure. We present a rare case of a 40-year-old male who presented to our emergency department with complaints of hematemesis and Malena and was later diagnosed with NCPH associated with chronic myeloid leukemia (CML). This case report emphasizes the importance of considering rare causes of NCPH like CML while evaluating such cases.

## Introduction

Portal hypertension (PH) is most commonly seen in the context of liver cirrhosis. Non-cirrhotic portal hypertension (NCPH) can, nevertheless, be a complication of a variety of systemic, predominantly extrahepatic conditions. In the absence of cirrhosis, NCPH is characterized as a condition with a portocaval pressure gradient greater than 5 mm Hg. Based on the site of vascular resistance, the various intra- and extrahepatic causes of NCPH can be divided into prehepatic, hepatic (presinusoidal, sinusoidal, and post-sinusoidal), and post-hepatic variants. The leading cause of NCPH worldwide is schistosomiasis; however, a wide range of systemic illnesses, from infections to malignancies, can also result in the development of NCPH [1]. The development of portal hypertension is thought to occur via a variety of pathophysiological mechanisms, including increased vascular resistance (sinusoidal compression, sinusoidal occlusion/infiltration, vascular remodeling, and venous thrombosis), as well as increased flow through arterioportal shunts. Portal hypertension has been linked to several different pathways, including portal vein damage, congenital abnormalities, and hypercoagulable conditions, among others. Gene mutations or acquired conditions can both result in hypercoagulable states [2]. Oral contraceptive use, pregnancy, and myeloproliferative diseases (MPDs) are some examples of acquired causes. MPDs, both latent and occult, are known to be a significantly rare cause of portal hypertension [3,4]. We present a rare case of a 40-year-old male with chronic myeloid leukemia (CML) presenting with non-cirrhotic portal hypertension.

## Case presentation

A 40-year-old male patient presented to the emergency department with the chief complaints of two to three episodes of hematemesis and Malena in the previous two days. He also complained of a loss of appetite and generalized weakness for the last 15 days.

He denied any history of hypertension, diabetes mellitus, ischemic heart disease, tuberculosis, and thyroid disorder. No history of tobacco smoking or alcohol intake. The vital signs were normal. On examination, the patient had icterus and pallor. An abdominal examination revealed splenomegaly without a sign of fluid. Cardiovascular and respiratory system examinations were normal.

**Figure 1:**
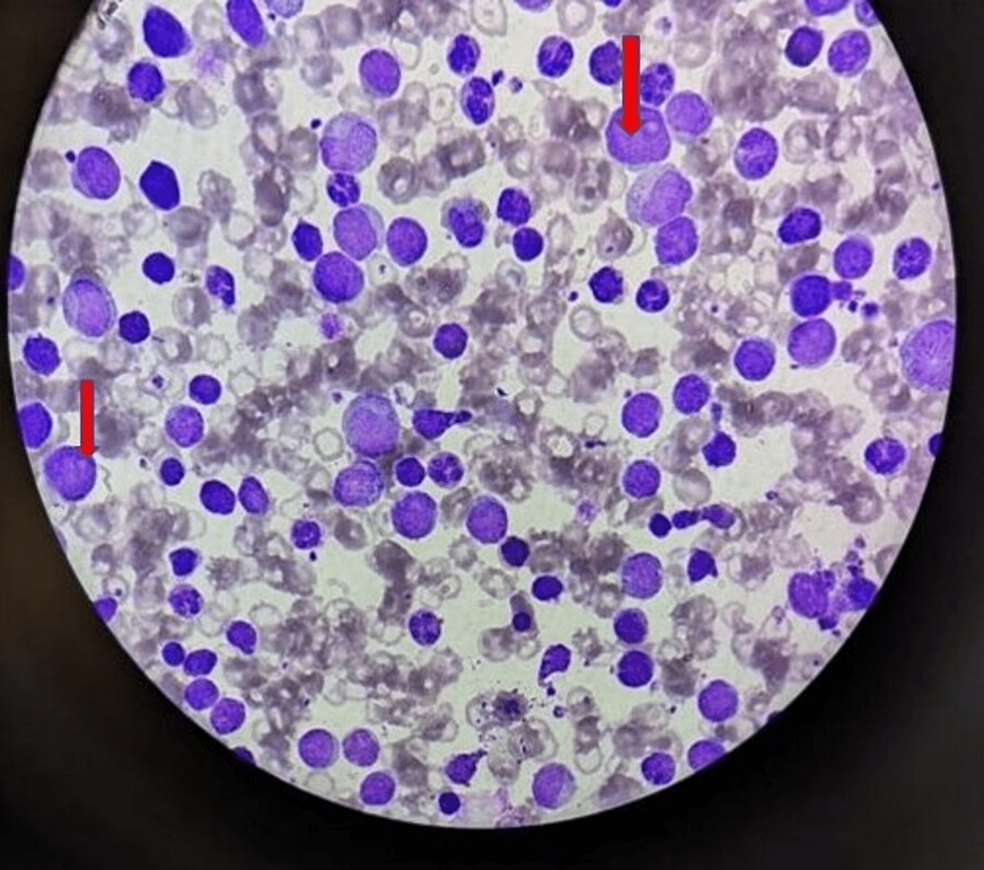
Peripheral smear showing myeloblast cells (red arrow).

On admission, the laboratory values are highlighted in Table 1. Peripheral smear was suggestive of microcytic hypochromic red blood cells with anisopoikilocytosis showing few pencil cells, tear drop cells, and blast cells, as shown in Figure 1.

**Table 1:** Laboratory parameter of the patient with reference range.

Lab parameters	Observed value	Normal range
Hemoglobin	6.2 gm% (low)	13–17 gm%
Mean corpuscular volume	71.8 fL (low)	83–101 fL
Total leucocyte count	2,52,400 cells/cu mm (high)	4000–10,000 cells/cu mm
Platelets	1.32 lakhs/cu mm (normal)	1.5–4.1 lakhs/cu mm
Urea	30 mg/dL (normal)	19–43 mg/dl
Creatinine	0.9 mg/dL (normal)	0.66–1.25 mg/dL
Sodium	132 mmol/L (low)	137–145 mmol/l
Potassium	4.5 mmol/l (normal)	3.5–5.1 mmol/L
Alkaline phosphatase	417 U/L (high)	38–126 U/L
Alanine transaminase	19 U/L (normal)	<50 U/L
Aspartate transaminase	22 U/L (normal)	17–59 U/L
Albumin	2.4 g/dL (low)	3.5–5 g/dL
Total bilirubin	1.9 mg/dl (high)	0.2–1.3 mg/dl
Activated partial thromboplastin time	38.0 (high)	29.5–31.0
Prothrombin time	32.3 (high)	11.9–13
International normalized ratio	2.88 (high)	0.9–1.4

**Figure 2:**
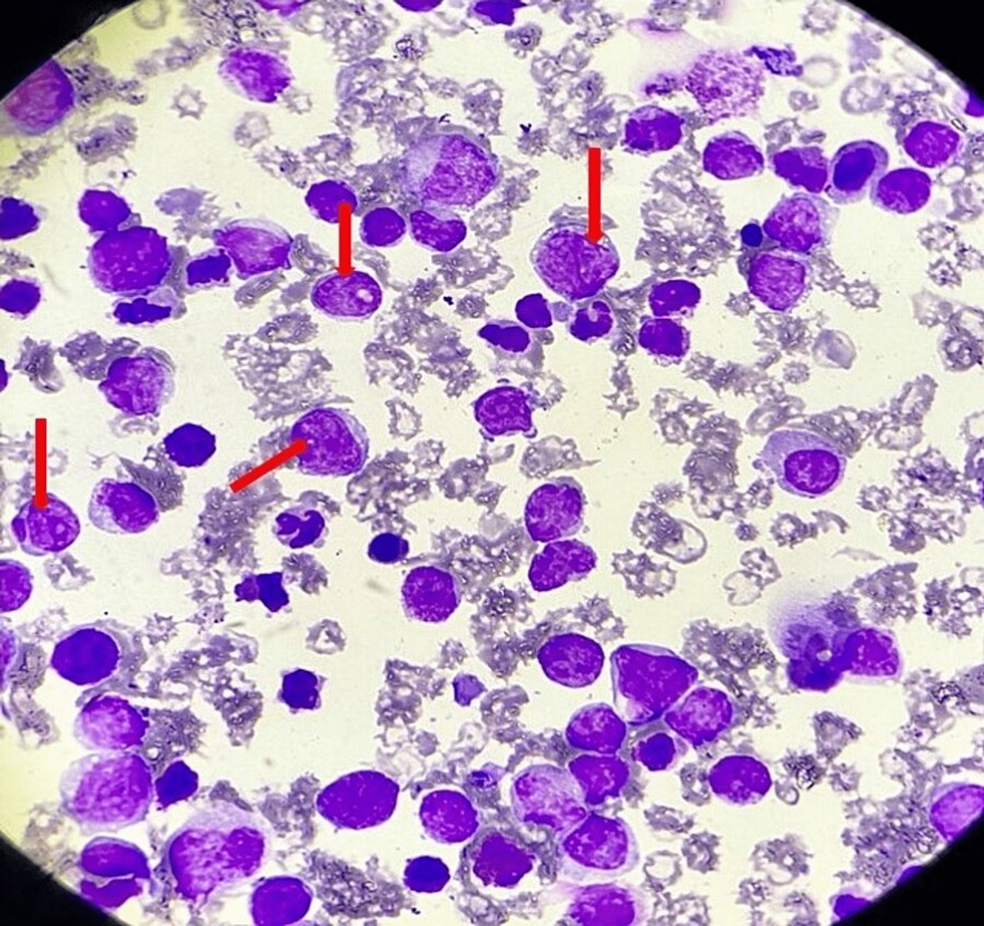
Bone marrow showing myeloblast cells (red arrow).

No hemoparasite was seen. Chronic myeloid leukemia in the accelerated phase was considered based on clinical and laboratory parameters. Bone marrow aspiration cytology was performed, revealing blast cells up to 57% with a marked increase in myeloid to erythroid ratio, indicating CML in blast crisis, as shown in Figure 2.

**Figure 3:**
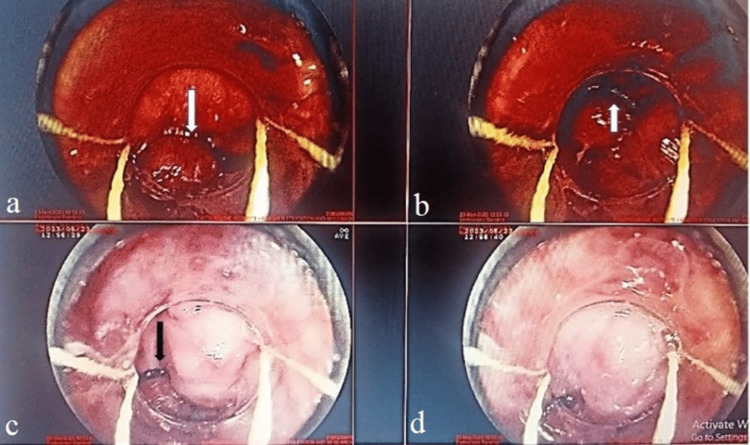
Bleeding varices (white arrow); (a) and (b) banding (black arrow); (c) and (d) demonstrate variceal banding.

Upper GI endoscopy revealed large esophageal varices, and banding was done as shown in Figure 3. Based on clinical history, past history, examination, and investigations non-cirrhotic portal hypertension due to CML was considered.

**Figure 4:**
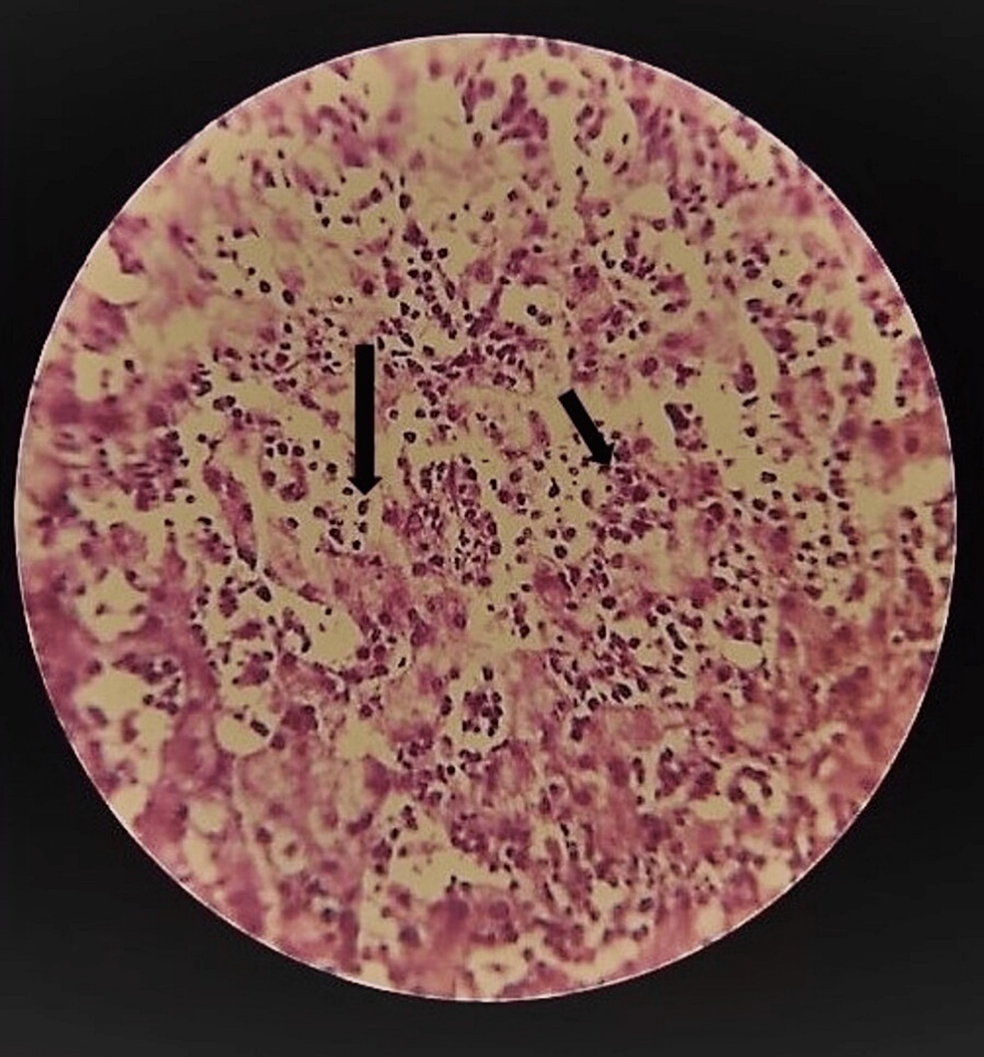
Liver biopsy showing infiltrating blast cells (black arrow) surrounded by hepatocytes.

Contrast-enhanced computed tomography of the abdomen revealed hepatomegaly with pre-stenotic portal vein dilatation/aneurysm, metallic clips in-situ, and multiple portosystemic collaterals, indicating the diagnosis of non-cirrhotic portal hypertension. A liver biopsy revealed intrahepatic cholestasis, cholangitis, and lymphocyte and myeloid series cell infiltrating canaliculi, supporting the diagnosis of CML as shown in Figure 4. Given the high probability of CML, his genetic study was done, which found a positive translocation (9;22) (q34; q11.2) for the breakpoint cluster region-Abelson (BCR-ABL) Philadelphia gene by fluorescence in-situ hybridization (FISH) and a positive JAK 2 (exon 14) mutation. As the patient was in the chronic phase of CML with Philadelphia, he was started on tablet imatinib 400 mg thrice daily. On follow-up, the patient’s condition significantly improved in terms of biochemical parameters, such that his total leucocyte counts were reduced to 12,456 cells/cumm after two months.

## Discussion

Non-cirrhotic portal hypertension (NCPH) is a poorly known condition that manifests as portal hypertension without the presence of traditional hepatic cirrhosis, which is characterized by widespread nodules completely encircled by fibrous septa [4,5]. It has been linked to immunological diseases, exposure to toxic substances or medications, myeloproliferative neoplasms, and blood coagulation abnormalities. Most instances begin with intrahepatic portal venous thrombosis or sclerosis, with varying degrees of prehepatic portal system involvement. The exact causes of PH in CML are still unknown [6]. Two explanations have been offered in the absence of portal and/or hepatic vein thromboses. According to the first theory, PH occurs in CML patients as a result of sinusoidal narrowing and intrahepatic obstruction because of extramedullary hematopoiesis and myeloid cell infiltration of the liver, which increases intrahepatic resistance. As per the second theory, PH occurs in such patients as a result of increased portal blood flow through the enlarged spleen. One of the main causes of PH in hematological disorders is portal vein thrombosis. It is unclear if portal vein thrombosis in CML is induced by a hypercoagulable state and hyperviscosity caused by the underlying disease or by stasis caused by elevated sinusoidal pressure [7,8]. Our patient had an extremely high white blood cell count (252,400 cells/mL), which may have contributed to thrombosis by increasing blood viscosity. In the absence of thrombosis, PH has been associated with an increase in intrahepatic resistance and sinusoidal narrowing due to myeloid metaplasia, as well as a considerable increase in portal flow due to splenomegaly. There have been few reports of PH in patients with myelofibrosis due to increased splenic and/or portal flow accompanied by reduced hematopoiesis. Some case reports demonstrated experimentally that PH does not develop as a result of increased portal flow [8]. In such cases, the absence of structural alterations in the liver and elevated intrahepatic resistance produced by obstruction from extramedullary hematopoiesis of the liver was the key causative factor. Liver biopsy, in our case, demonstrated infiltration of liver sinusoids with hematopoietic cells and myeloid metaplasia considering it as a possible explanation and important mechanism in the development of non-cirrhotic portal hypertension.

## Conclusions

The pathophysiological mechanisms underlying NCPH in CML remain unclear. Our case highlights the importance of considering rare possibilities like CML as a potential cause of NCPH in similar clinical scenarios. The diagnosis of NCPH associated with CML requires a comprehensive evaluation and a multidisciplinary approach involving hematologists, gastroenterologists, and pathologists to provide an accurate diagnosis and appropriate management. Treatment options for NCPH associated with CML include targeted therapy, such as the use of tyrosine kinase inhibitors like imatinib. Regular follow-up and monitoring of the patient's condition are essential to assess the response to treatment and ensure optimal outcomes. Further research and studies are warranted to enhance our understanding of the pathogenesis and management of NCPH associated with CML, ultimately leading to improved outcomes for affected patients.

## References

[1] Schouten JN, Garcia-Pagan JC, Valla DC, Janssen HL (2011). Idiopathic noncirrhotic portal hypertension. Hepatology.

[2] Semela D (2015). Systemic disease associated with noncirrhotic portal hypertension. Clin Liver Dis (Hoboken).

[3] Bodh V, Chawla Y (2014). Noncirrhotic IPH. Clin Liver Dis (Hoboken).

[4] Malik S, Wadekar A, Acharya S (2021). Porto pulmonary hypertension (POPH) in a female with non-cirrhotic portal hypertension-early suspicion and early diagnosis is the key. J Evol Med Dent Sci.

[5] Khanna R, Sarin SK (2014). Non-cirrhotic portal hypertension-diagnosis and management. J Hepatol.

[6] Kumar S, Joshi R, Jain AP (2007). Portal hypertension associated with sickle cell disease. Indian J Gastroenterol.

[7] Toros AB, Gokcay S, Cetin G, Ar MC, Karagoz Y, Kesici B (2013). Portal hypertension and myeloproliferative neoplasms: a relationship revealed. ISRN Hematol.

[8] Abu-Hilal M, Tawaker J (2009). Portal hypertension secondary to myelofibrosis with myeloid metaplasia: a study of 13 cases. World J Gastroenterol.

